# Expanding the clinical phenotype of the 3q29 microdeletion syndrome and characterization of the reciprocal microduplication

**DOI:** 10.1186/1755-8166-1-8

**Published:** 2008-04-28

**Authors:** Blake C Ballif, Aaron Theisen, Justine Coppinger, Gordon C Gowans, Joseph H Hersh, Suneeta Madan-Khetarpal, Karen R Schmidt, Raymond Tervo, Luis F Escobar, Christopher A Friedrich, Marie McDonald, Lindsey Campbell, Jeffrey E Ming, Elaine H Zackai, Bassem A Bejjani, Lisa G Shaffer

**Affiliations:** 1 Signature Genomic Laboratories, LLC, Spokane, WA, USA; 2 Weisskopf Child Evaluation Center, Department of Pediatrics, University of Louisville, Louisville, KY, USA; 3 Children's Hospital of Pittsburgh, Pittsburgh, PA, USA; 4 Gillette Children's Specialty Healthcare, St. Paul, MN, USA; 5 St. Vincent Hospital, Indianapolis, IN, USA; 6 Department of Preventive Medicine, University of Mississippi Medical Center, Jackson, MS, USA; 7 Division of Medical Genetics, Duke University Medical Center, Durham, NC, USA; 8 The Children's Hospital of Philadelphia, Philadelphia, PA, USA; 9 School of Molecular Biosciences, Washington State University, Spokane, WA, USA; 10 Sacred Heart Medical Center, Spokane, WA, USA

## Abstract

**Background:**

Interstitial deletions of 3q29 have been recently described as a microdeletion syndrome mediated by nonallelic homologous recombination between low-copy repeats resulting in an ~1.6 Mb common-sized deletion. Given the molecular mechanism causing the deletion, the reciprocal duplication is anticipated to occur with equal frequency, although only one family with this duplication has been reported.

**Results:**

In this study we describe 14 individuals with microdeletions of 3q29, including one family with a mildly affected mother and two affected children, identified among 14,698 individuals with idiopathic mental retardation who were analyzed by array CGH. Eleven individuals had typical 1.6-Mb deletions. Three individuals had deletions that flank, span, or partially overlap the commonly deleted region. Although the clinical presentations of individuals with typical-sized deletions varied, several features were present in multiple individuals, including mental retardation and microcephaly. We also identified 19 individuals with duplications of 3q29, five of which appear to be the reciprocal duplication product of the 3q29 microdeletion and 14 of which flank, span, or partially overlap the common deletion region. The clinical features of individuals with microduplications of 3q29 also varied with few common features. *De novo *and inherited abnormalities were found in both the microdeletion and microduplication cohorts illustrating the need for parental samples to fully characterize these abnormalities.

**Conclusion:**

Our report demonstrates that array CGH is especially suited to identify chromosome abnormalities with unclear or variable presentations.

## Introduction

Interstitial deletions of 3q29 have recently been described as a new microdeletion syndrome [1]. Eight cases have been reported in the literature [1-3]. Although the deletion size is the same in all cases studied (1.6 Mb), the phenotype is variable, with mild to moderate mental retardation the only feature common to all individuals. However, most individuals exhibit mildly dysmorphic features, including a long, narrow face, short philtrum, high nasal bridge, and large ears. Speech delay, autistic traits, chest-wall deformities, ungainly or ataxic gait, and long and tapering fingers were each described in at least two individuals. Several anomalies, such as microcephaly, cleft lip and palate, recurrent middle ear infections, ligamentous laxity, abnormal skin pigmentation, horseshoe kidney and hypospadias, have been described in individual patients.

The 3q29 interstitial deletion region encompasses five known genes and at least 17 transcripts with unknown functions and is flanked by two highly homologous region-specific low-copy repeats (LCRs) [1]. The presence of LCRs flanking the deletions in all subjects suggests that microdeletions of 3q29 arise via nonallelic homologous recombination (NAHR) between LCRs on either side of the breakpoint [4,5]. LCRs flank a number of rearrangement "hotspots." The most well-studied region prone to rearrangement is 17p12; 24-kb repeats flank a 1.5-Mb region, deletion of which results in hereditary neuropathy with liability to pressure palsies (HNPP), whereas duplication of the same region results in Charcot-Marie-Tooth disease type 1A [6]. If NAHR between flanking LCRs is indeed the mechanism underlying deletions of 3q29, reciprocal duplications of the same interval would be expected to occur with equal frequency. Recently a three-generation family was identified in which five individuals had microduplications that overlapped the region typically deleted in 3q29 microdeletion syndrome [7]. Common clinical features included mild/moderate mental retardation and microcephaly.

In our diagnostic laboratory we have analyzed 14,698 cases by array CGH using a microarray which includes a high resolution, near-tiling-path coverage of the entire 5.7 Mb of 3q29. Herein, we report the identification of 14 individuals with microdeletions of 3q29 in addition to 19 individuals with duplications of 3q29, five of which appear to be the reciprocal duplication product of the 3q29 microdeletion and 14 with duplications that flank, span, or partially overlap the common deletion region.

## Subjects and Methods

### Patients and controls

During the period encompassing March 2004 through September 2007, we screened 14,698 consecutive individuals with developmental disabilities whose clinical specimens were submitted to our laboratory from the United States and abroad. The most common clinical presentations of the individuals referred for testing were mental retardation, developmental delay, or multiple congenital anomalies. Most subjects had previous normal cytogenetic analysis, subtelomere FISH, and/or locus-specific FISH. For the individuals with 3q29 abnormalities described here, informed consent was obtained using a Signature Genomic Laboratories consent form to perform high-resolution molecular cytogenetic testing and to publish photographs.

### BAC Array CGH

Array CGH was performed with a bacterial artificial chromosome (BAC) microarray (the SignatureChip^®^; Signature Genomic Laboratories, Spokane, WA) that was developed for the detection of microdeletions, microduplications, aneuploidy, unbalanced translocations, and subtelomeric and pericentromeric copy-number alterations [8]. The current version of the SignatureChip, the SignatureChip Whole Genome™ (SignatureChipWG), contains 4670 BACs representing 1543 loci with each locus being represented by a minimum of three overlapping clones. The subtelomeric and pericentromeric regions are represented with a higher density of overlapping BAC clones, targeted to the unique sequences adjacent to these repetitive regions and consisting of contigs of clones located approximately every 0.5 Mb spanning more than 5 Mb. Genes in important developmental pathways are also covered by contigs of BACs to fill in the chromosome arms and provide higher resolution with an average gap size between contigs of ~1.6 Mb [8].

Microarray analysis was performed as described [8], with the following modifications: Briefly, genomic DNA was extracted from peripheral blood using a Qiagen M48 Biorobot automated DNA extraction system. Purified genomic DNA was then sonicated and labeled with Alexaflour dyes 555 or 647 using a BioPrime Total DNA labeling kit (Invitrogen Corp). Microarrays were hybridized as previously described (8) and washed using a Little Dipper automated microarray washing station (SciGene). Microarrays were scanned on an Axon 4000B scanner (Molecular Devices) and signal intensity ratios were analyzed as described (8) using a custom analysis and display interface (Genoglyphix™).

### FISH

All abnormalities detected by array CGH were confirmed and visualized by metaphase or interphase fluorescence *in situ *hybridization (FISH) using one or more BAC clones determined to be abnormal by array CGH [9].

### Oligonucleotide Array CGH

Oligonucleotide array CGH was performed using a custom-designed microarray (Agilent Technologies, Santa Clara, USA) on 30 samples in which an abnormality of 3q29 was detected by BAC array CGH. The microarray consisted of ~13,000 60-mer oligonucleotide probes spanning ~5.7 Mb of 3q29, with an average probe spacing of 430 bp. Regions of 3q29 not represented by oligonucleotides on the high-density 3q29 microarray consist primarily of regions of large segmental duplication, such as LCRs, or gaps in the human genome sequence assembly. However, the lack of coverage in some of these regions was mitigated by the placement of oligos within a few hundred basepairs of the LCRs.

Genomic DNA labeling was performed as described for BAC arrays whereas array hybridization and washing was performed as specified by the manufacturer (Agilent Technologies) for 8-plex CGH microarrays. Arrays were scanned using an Axon 4000B scanner (Molecular Devices) and analyzed using Agilent Feature Extraction software v9.5.1 and Agilent CGH Analytics software v3.5.14.

## Results

**Figure 1:**
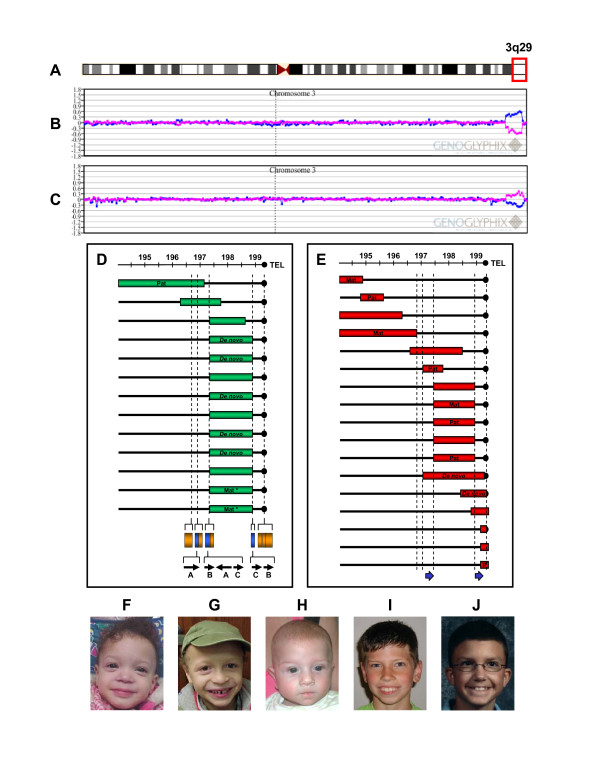
Summary of array CGH results on individuals with microdeletions and microduplications of 3q29. **(A) **Ideogram of chromosome 3 showing the location of the 3q29 cytogenetic band. **(B) **Representative SignatureChipWG result plot for an individual with a single copy loss of 11 BAC clones spanning the entire 1.6 Mb common microdeletion region. Each clone represented on the array is arranged along the x-axis according to its location on chromosome 3 with the most distal/telomeric p-arm clones on the left and the most distal/telomeric q-arm clones on the right. The blue line represents the ratios for each clone from the first experiment (control/patient), and the pink line represents the ratios for each clone obtained from the second experiment in which the dyes have been reversed (patient/control). **(C) **Representative SignatureChipWG result plot for an individual with a single copy gain of 11 BAC clones spanning the entire 1.6 Mb common microdeletion region. **(D) **Diagram showing the deletion sizes of 13 individuals with microdeletions of 3q29 as refined using the high-density, custom 3q29 oligonucleotide microarray. Vertical dashed lines in D and E indicate the location of the low-copy repeats (LCRs) in the 3q29 interval for which oligonucleotides were not included on the microarray. Blue bars shown at the bottom of the diagram indicate the location of paired LCRs within 3q29; their precise orientation is illustrated by arrows. Orange bars indicate the location of other segmental duplications that share no homology with any other regions on chromosome 3. LCRs A, B, and C are ~40 kb, ~20 kb, and ~20 kb long, respectively, with > 90% identity to their paired counterparts and are simplified for illustrative purposes (based on the Segmental Duplications Database, May 2004 (hg17) draft). **(E) **Diagram showing the duplication sizes of 17 individuals with microduplications of 3q29 as refined using the high-density, custom 3q29 oligonucleotide microarray. Blue arrows show the location of the LCRs that mediate the common 1.6 Mb microdeletion/microduplication and are not drawn to scale. **(F-J) **Photographs of five of the 11 individuals with common-sized microdeletions of 3q29. Individuals shown in F and G are siblings.

We identified 14 individuals with microdeletions of 3q29 amongst our patient population, including one family with a mildly affected mother and two affected children (Fig. 1B). We characterized 13 of the 14 deletions in more detail using a high-density oligonucleotide microarray (insufficient DNA was available for testing of the mother of the two affected children). Eleven individuals had typical 1.6 Mb deletions. Both BAC and oligo arrays confirmed that these eleven deletions had breakpoints flanked by segmental duplications known to mediate the common-sized microdeletion of 3q29 (Fig. 1D). Of the other deletions, one ~1.4 Mb deletion was within the common deletion region with only its proximal breakpoint flanked by LCRs, one ~1.5 Mb deletion overlapped the proximal end of the common deletion region by ~500 kb and extended more proximally, and one deletion > 3.2 Mb in size flanked the proximal end of the commonly deleted region (Fig. 1D). Neither the 1.5 Mb deletion nor the 3.2 Mb deletion has breakpoints flanked by LCRs. We confirmed a deletion in all 14 individuals by FISH. Of the eight individuals for whom parental samples were available for analysis, five had *de novo *abnormalities, all of which were the common-sized deletion. FISH also identified a deletion of 3q29 in the mother of the two siblings with microdeletion of 3q29. BAC microarray analysis of the father of the individual with the > 3.2 Mb proximal deletion showed the same deletion, indicating that the patient's deletion was paternally derived.

Clinical information was available for seven individuals with the typical 1.6 Mb deletion (Table 1). The clinical features common to three or more individuals in our cohort include microcephaly or small head, large low-set and posteriorly rotated ears, wide nasal bridge, and language delay. High-arched palate, widely spaced teeth, clumsy gait, head banging, macrocephaly, patent ductus arteriosus, chest cavity deformity, and hypospadias were identified in one or two individuals (Table 1).

**Table 1:** Summary of clinical features found in individuals with common-sized 3q29 microdeletion in this and previous studies.

Feature	Number of individuals in present study with feature	Number of individuals in previous reports with feature	Number of individuals reported to date with feature/Number of individuals reported to date^a^
Mild/moderate mental retardation	7	8	15/15
Long, narrow face	0	3	3/15
Short philtrum	0	6	6/15
High nasal bridge	4	5	9/15
Large, low-set, posteriorly rotated ears	4	1	5/15
Speech delay	3	5	8/15
Delayed walking	2	5	7/15
Autism/autistic features	1	3	4/15
Chest-cavity deformities	1	2	3/15
Ataxic gait/gait abnormalities	2	3	5/15
Long, tapering fingers	0	3	3/15
Microcephaly^a^	5	2	7/15
Macrocephaly	1	0	1/15
Cleft lip/palate	0	1	1/15
High-arched palate	2	0	2/15
Widely spaced teeth	2	0	2/15
Recurrent middle ear infections	1	1	2/15
Ligamentous laxity	0	1	1/15
Abnormal skin pigmentation	0	1	1/15
Horseshoe kidney	0	1	1/15
Hypospadias	1	1	2/15
Nasal voice	1	1	2/15
Six lumbar vertebrae	0	1	1/15
Cerebral sigmoid venous thrombosis	0	1	1/15

^a ^Includes previous reports [1-3] and patients reported in this paper; ^b ^includes reports of progressive microcephaly

We also identified 19 individuals with microduplications of 3q29 (Fig. 1C). We analyzed 17 of the 19 microduplications of 3q29 using the high-density 3q29 oligo array to refine the breakpoints (Fig. 1E). The high-resolution microarray identified 12 different duplication sizes that range in size from 200 kb to 2.4 Mb and which flank, span, or partially overlap the commonly deleted region. We also identified five cases which appear to be the reciprocal duplication product of the 3q29 microdeletion because they are flanked by the LCRs which mediate the common microdeletion of 3q29. Although five other microduplications of 3q29 have one breakpoint that is flanked by an LCR, only the reciprocal duplications have both breakpoints flanked by LCRs (Fig. 1E).

For the 10 individuals with 3q29 microduplications for whom parental DNA was available for microarray analysis, two had *de novo *abnormalities. The three reciprocal 3q29 duplications for which parental samples were available for testing were all inherited from a parent.

Clinical information was available for seven individuals with microduplications of 3q29, three of whom had the reciprocal product of the common-sized deletion. The only clinical feature common to these three individuals was mild to moderate mental retardation (Table 2). Among individuals with microduplications of different sizes, craniosynostosis, high palate, seizures, and ventricular septal defect were each identified twice.

**Table 2:** Summary of clinical features found in individuals with common-sized 3q29 microduplication in this and previous studies.

Feature	Number of individuals in present study with feature	Number of individuals in previous study^a ^with feature	Number of individuals reported to date with feature/Number of individuals reported to date
Mild/moderate mental retardation	3	3^b^	6/8
Craniosynostosis	1	0	1/8
Macrocephaly	1	0	1/8
Microcephaly	1	4	5/8
High palate	1	0	1/8
Ventricular septal defect	1	0	1/8
Excessive hand creases	0	2	2/8
Pes planus	0	2	2/8
Obesity	1	3	4/8

^a ^[ref. 7] round face and bulbous nose were also noted, but these traits may be familial; ^b^three of five family members were old enough for testing

## Discussion

High-resolution microarray analysis has facilitated the identification of multiple new microdeletion/microduplication syndromes in individuals with idiopathic mental retardation and congenital anomalies [10-13]. Because it is not reliant on clinical suspicion of a known syndrome, microarray analysis can identify the underlying genomic lesion in affected individuals for whom a diagnosis has not been possible – because the rarity of the causative chromosomal abnormality may prevent accurate reporting of index cases in the literature or because the variability or mildness of the phenotype escapes characterization.

Among individuals with microdeletions of 3q29, the phenotype varies widely, with mild to moderate mental retardation/developmental delay, microcephaly, and mild dysmorphic features (including high nasal bridge and short philtrum) the only features common to the majority. The lack of an easily discernible clinical phenotype complicates diagnosis and may explain why so few individuals with this microdeletion have been identified in the literature. However, the presence of autistic features in several individuals [1,2] suggests that a deletion of 3q29 may be considered in individuals with autism. Like that of the 3q29 microdeletion, the phenotype of the reciprocal duplication varied, with mental retardation the only common feature among the three individuals for whom clinical information was available. The careful clinical examination of additional individuals with these chromosome abnormalities are needed to further refine the phenotypes.

Although microduplications have been predicted to occur with equal frequency as microdeletions that are flanked by LCRs, fewer have been identified. Those that have been found tend to result in milder phenotypes than microdeletions, which may explain in part why microduplication syndromes appear to be less common than their counterparts. Of the 19 microduplications of 3q29 identified by our laboratory, only five were the reciprocal duplication of the common-sized deletion of the same region; the remaining 14 microduplications flanked, overlapped or were smaller than the common deletion region. These results suggest that other mechanisms in addition to NAHR mediate rearrangements of 3q29. A similar situation occurs in rare rearrangements of 17p11.2 associated with Smith-Magenis syndrome, in which the breakpoints do not fall within the paired segmental duplications that flank most deletions of this region or appear to be mediated by known genomic architectural features [14].

Only two of 10 individuals with microduplications of 3q29 for whom parental DNA was available for testing had *de novo *abnormalities. Typically, abnormalities inherited from phenotypically normal parents are considered benign copy-number variants. However, subclinical phenotypes not appreciated in a parent may be manifested more severely in an affected child; the most common example is the deletion of 22q11.2 [15]. Therefore, the clinical significance of the reciprocal 3q29 microduplication is still unclear. The three-generation family with five individuals with reciprocal 3q29 microduplications recently described by Lisi and colleagues [7] would seem to confirm this duplication is a clinically relevant abnormality. However, the presence of a ~169 kb deletion of 4q13.1 in all affected individuals, and absent in unaffected family members, suggests that the 3q29 microduplication may not be causative of the affected individuals' phenotypes. Nonetheless, given the rate at which de novo genomic rearrangements occur in the human population, a combination of CNVs at the same or different loci, inherited from parents in whom the single variation was insufficient to cause disease, might be necessary to produce a phenotype. A similar hypothesis has been proposed for thrombocytopenia-absent radius (TAR) syndrome; the authors of a recent study in which 30 individuals with TAR syndrome had a microdeletion of 1q21.1, 75% of which were inherited from a normal parent, suggest the presence of a genetic modifier in addition to the 1q21.1 microdeletion was necessary to cause the disease phenotype [16]. High-resolution whole-genome screening of a large cohort of affected individuals is required to determine whether another abnormality is present elsewhere in the genome.

High-resolution breakpoint mapping using BAC and oligo-based microarrays identified common and unique breakpoints in both the 3q29 microdeletion and microduplication cohorts suggesting that other mechanisms in addition to nonallelic homologous recombination may play a role in mediating these rearrangements. This study demonstrates that array CGH is especially suited to identify chromosome abnormalities in individuals with unclear or variable presentations.

## Competing interests

BC Ballif, A Theisen, and J Coppinger are employees of Signature Genomic Laboratories, LLC. BA Bejjani and LG Shaffer have ownership, receive consulting fees, and sit on the Members' Board of Signature Genomic Laboratories, LLC.

## Consent statement

Informed consent to perform high-resolution molecular cytogenetic testing and to publish photographs was obtained from subjects' parents or legal guardians using a Signature Genomic Laboratories consent form.

## Authors' contributions

BCB conceived of the study and carried out the molecular genetic studies; AT drafted the manuscript; JC coordinated the ascertainment of clinical information from the referring physicians; GCC, JHH, SM-K, KRS, RT, LFE, CAF, MM, JEM, and EHZ referred patients for the study and contributed clinical information; BAB and LGS coordinated the study. All authors have read and approved the manuscript.
